# Enucleation and development of cluster headache: a retrospective study

**DOI:** 10.1186/1471-2377-5-6

**Published:** 2005-03-22

**Authors:** Peter Sörös, Oanh Vo, Heinrich Gerding, Ingo W Husstedt, Stefan Evers

**Affiliations:** 1 Department of Neurology, Münster University Hospital, Albert-Schweizer-Strasse 33, 48149 Münster, Germany; 2 Department of Ophthalmology, Münster University Hospital, Domagkstrasse 15, 48149 Münster, Germany; 3 Department of Imaging Research, Sunnybrook and Women's College Health Sciences Centre, 2075 Bayview Avenue, Toronto, Ontario, M4N 3M5, Canada

## Abstract

**Background:**

Cluster headache (CH) is a neurovascular, primary headache disorder. There are, however, several case reports about patients whose CH started shortly after a structural brain disease or trauma. Motivated by a patient who developed CH 3 weeks after the removal of an eye and by similar case reports, we tested the hypothesis that the removal of an eye is a risk factor for CH.

**Methods:**

A detailed headache questionnaire was filled out by 112 patients on average 8 years after enucleation or evisceration of an eye.

**Results:**

While 21 % of these patients experienced previously unknown headaches after the removal of an eye, no patient fulfilled the diagnostic criteria for CH.

**Conclusion:**

Our data does not suggest that the removal of an eye is a major risk factor for the development of CH.

## Background

**Figure 1:**
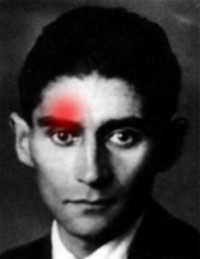
**Pain in cluster headache**. The location of intense pain, usually orbital or supraorbital, in a CH attack, overlayed onto the portrait of Franz Kafka (1924). Kafka suffered from extremely severe headache attacks, possibly CH [39].

Cluster headache (CH) is characterized by severe attacks of unilateral pain and cranial autonomic dysfunction [1]. The pain is usually felt in and around the orbita or adjacent areas of the head (Fig. 1). CH attacks last 15–180 minutes and occur, during a cluster period, from once every other day to several times a day. The symptoms of autonomic dysfunction are always ipsilateral to the pain and can involve the eye (conjunctival injection, lacrimation, miosis, ptosis, eyelid edema), the nose (nasal congestion, rhinorrhea), and the face (sweating) [1]. The exact pathophysiology of CH is, more than 250 years after the first description of episodic CH by Gerhard van Swieten in 1745 [2], still unresolved. Several convergent lines of evidence suggest that CH is a primary neurovascular disorder (for review see [3]). The hypothalamus is supposed to be involved in the pathogenesis of CH and might even be pivotal for the timing of cluster periods and single attacks [4].

Challenging the notion of an exclusively primary headache disorder, several reports described the onset of CH during or shortly after traumatic brain injury [5,6] or structural brain disease (reviewed in [7,8]). In many of these reports, vascular disorders (e.g. arterio-venous malformations [9,10], aneurysms [11], dissections [12], or fistulas [13] of the cranial vasculature) were associated with the onset of CH. In other patients, intracranial neoplasms (e.g. meningeomas [14], adenomas [15]) or local inflammatory processes [16,17] were seen in connection with the beginning of CH. All reports on putative secondary CH face the difficult question whether there is a causal relationship or a mere coincidence between CH and the preceding brain injury or disease. The International Classification of Headache Disorders suggests to code a headache with the characteristics of a primary headache (e.g. CH) as a secondary headache if it occurs for the first time and in close temporal relation to another disorder that is a known cause of headache [1]. We follow the phenomenologic classification of the International Headache Society (IHS) [18] and regard all headaches with the symptomatology of CH and developing in a close temporal relationship to a brain injury or disease as secondary headaches.

In addition to the aforementioned patients, we saw a 37-year-old man who fulfilled the IHS criteria for a secondary CH. He developed strictly right-sided episodic CH 3 weeks after the removal of his right eye bulb [19]. The patient not only fulfilled the diagnostic criteria for CH [1], but also responded to standard acute and prophylactic CH therapy. At least another 5 patients, all of them male, could be identified in the literature who developed CH, either episodic or chronic, after the removal of the eye bulb [19]. Episodic CH originated in a 45-year-old man one year after maxillectomy and exenteration due to a squamous cell carcinoma [20]. In other patients, however, CH developed up to 18 years after surgery [21]. Despite the suggestive chronology in our patient with a latency between surgery and first CH attack of only 3 weeks, it remained unclear if the removal of the eye influenced the development of CH or if both events were unrelated. To test the hypothesis that removal of an eye is a risk factor for CH we conducted a retrospective survey among individuals whose eye had to be resected.

## Methods

To identify patients who underwent the removal of an eye, the clinical records of all inpatients admitted to the Department of Ophthalmology, Minister University Hospital between 1986 and 1995 were reviewed. In total, 332 patients had enucleation or evisceration of one eye in the specified time frame. All patients received a detailed questionnaire via mail asking about the occurrence and clinical presentation of headaches before and after the removal of the bulb (33 questions in total). The questionnaire was developed by the authors and included, amongst others, questions about the location, the quality, the duration of individual headache attacks, the occurrence of cluster periods, and the typical autonomic features of CH. The questionnaire included all diagnostic criteria for CH as described in the International Classification of Headache Disorders [1], but it was not formally validated. The patients were asked to return the completed questionnaire in a pre-paid envelope by mail. Medical records were consulted for personal data, the ophthalmologic diagnosis leading to the removal of the eye, and the operative technique used. A different aspect of this study regarding the prevalence and phenomenology of phantom experiences after removal of the eye was published previously [22]. The 37-year-old man who developed CH 3 weeks after removal of the ipsilateral eye, reported by us [19], was not included in the study population presented here because surgery was performed in an external hospital. A post-hoc analysis of statistical power was performed to assess the minimum effect size observable by the available sample size using the statistical package R for Mac OS X [23]. This analysis was based on the prevalence of CH found in two recent epidemiological studies [24,25]. The required statistical power was set to 0.8 and the significance level to 0.05.

## Results

One hundred twelve patients (78 men and 34 women) completed our questionnaire and were included in the data analysis (response rate, 33.7 %). The average age at the time of eye removal was 48 ± 21 years, and the average latency between eye removal and completing the questionnaire was 8 ± 3 years (range, 3–19 years). The major reasons for removal of the eye bulb were eye trauma (n = 40, 36 %), and malignant (n = 22, 20 %) or non-malignant eye diseases (n = 50, 44 %). Enucleation (removal of the globe with sparing of the extraocular muscles [26] was performed in 104 patients (93 %), and evisceration (removal of the contents of the globe with the sclera and the extraocular muscles left intact) was done in 8 patients (7 %). After the removal of the bulb, 24 patients (21 %) experienced previously unknown headaches. Although 13 of those patients reported strictly unilateral headaches (usually ipsilateral to the removed eye bulb), the characteristic autonomic symptoms of cluster headache and the typical temporal pattern of headache attacks were absent in all of those patients. None of the patients thus fulfilled the diagnostic criteria for CH [1]. The power analysis revealed that a CH prevalence of 5.5 or more in our group of 112 patients would have resulted in a significant increase of CH prevalence (based on a population-wide CH prevalence of 56/100,000 [25]). Based on a CH prevalence of 326/100,000 [24], a prevalence of 6.2 or more would have been significant.

## Discussion

**Figure 2:**
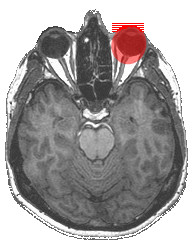
**Orbital anatomy**. High-resolution axial T1-weighted native MRI of a volunteer without structural abnormalities at 3.0 Tesla. Structures removed during enucleation (globe, part of the optic nerve, insertions of the external eye muscles) are highlighted by the red ellipse.

This retrospective study on 112 patients could not identify individuals who developed CH 3 – 19 years after removal of an eye bulb. During enucleation, the predominant technique used in our patients, the globe has to be separated from all orbital tissue, including the external eye muscles and the optic nerve (Fig. 2) [26]. The eye is innervated by the optic nerve, the nasociliary nerve and the sympathetic and parasympathetic fibers. This led us to the hypothesis that lesions of the autonomic network might induce secondary CH in patients after enucleation [19]. Similar mechanisms have been discussed in a patient with secondary CH due to an intracranial inflammatory pseudotumor in the posterior fossa [27]. Our result, however, does not provide evidence for the notion that a first CH attack after the removal of the bulb, as observed previously [19,20], is triggered or at least facilitated by the irritation of trigemino-autonomic nervous structures during surgery and wound healing. The techniques of evisceration and enucleation spare parts of the orbita (especially the external eye muscles) and usually do not affect the cranial nerves III, IV, and VI. During exenteration, on the other hand, the entire orbital content has to be removed. Patients with exenteration were not included in this study because this technique is used less frequently and mainly in cancer patients (which makes longer follow-up periods difficult). Patients with exenteration might be especially prone to develop CH because this procedure results in extensive damage of nervous tissue. As CH pain is often located in or around the orbita and the autonomic symptoms of CH usually involve the eye, it has been speculated if CH can occur without an ipsilateral eye. Patients developing ipsilateral CH after the removal of an eye [19] irrespective of the temporal relationship between these two events, clearly demonstrate that the eye and the surrounding tissues are not essential for the pathogenesis of CH.

CH usually develops between 20 and 45 years [28], with a mean age of onset of about 29 years [29,30]. CH, however, can start much later in life. Patients with a first CH attack at the age of 83 years and 75 years have been described [31]. As the mean age in our patients (48 years) is higher than the mean age of onset of CH, one might speculate that, in many of our patients, the removal of the eye was done when they were less susceptible for the development of CH. We do not suppose that the age difference between our sample of patients after eye removal and patients with first CH attack significantly influenced the result of this study. The reports on patients who developed CH in their 70ies and 80ies demonstrate that higher age does not prevent the development of CH. Moreover, many patients with secondary CH were older than the average age of onset of primary CH. The age of onset of the 5 patients with a first CH attack after removal of the eye was 36 years [19].

The presented study, however, has its limitations. The retrospective, questionnaire-based design might underestimate the prevalence of CH. Descriptions of the main symptoms of CH, however, either in questionnaires or letters, have proven to be useful in several epidemiological studies on CH [25,32-36] and take advantage of the distinct diagnostic criteria and the impressive nature of CH. The use of short self-adminstered questionnaires for screening larger populations has also been validated in migraine [37,38]. Our study was designed to screen our population via a questionnaire and then to verify each probable diagnosis of CH with a detailed interview and a comprehensive neurological examination (no patient, however, reported the main features of CH, short attacks of unilateral, severe headaches in bouts). Individuals with infrequent and mild bouts, however, might not recall past CH attacks while responding to a questionnaire. In addition, the relatively low response rate (34 %) might have influenced the present results. On the one hand, patients with CH might be hidden in the group of individuals not responding to our questionnaire. On the other hand, patients with CH are probably more willing to respond to a survey targeted at their symptoms than patients with other forms of headache or without headaches at all. In general, a prospective study which includes patients before the removal of an eye, follows them over years and then compares the outcome to a control group (i.e., a cohort study) would be more effective for the study of a disease's risk factors. In addition, CH is rare with a prevalence of less than 1 %. Investigating possible risk factors for rare disorders requires large sample or effect sizes. Our sample size is large enough to yield statistical significance for a CH prevalence of 5.5 % in our group (or 6.2 %, depending on the assumed population-wide CH prevalence), but might be too small to detect a slight increase of CH among patients after removal of an eye.

## Conclusion

Our data does not suggest that the removal of the globe is a major risk factor for the development of CH. We cannot exclude the possibility that enucleation is associated with a small increase in CH prevalence.

## Authors' contributions

All authors participated in designing the study and constructing the headache questionnaire. PS prepared the manuscript and performed the statistical analyses. OV contacted the patients, collected the data, and assisted with data analysis. HG participated in data collection, interpretation of the results, and drafting the manuscript. IWH and SE participated in interpretation of the results and in preparing the manuscript. All authors have read and approved the final manuscript.

## Pre-publication history

The pre-publication history for this paper can be accessed here:


